# Using Neurofeedback to Restore Inter-Hemispheric Imbalance: A Study Protocol for Adults With Dyslexia

**DOI:** 10.3389/fpsyg.2021.768061

**Published:** 2021-11-05

**Authors:** Alice Cancer, Maria Elide Vanutelli, Claudio Lucchiari, Alessandro Antonietti

**Affiliations:** ^1^Department of Psychology, Università Cattolica del Sacro Cuore, Milan, Italy; ^2^Department of Philosophy, Università degli Studi di Milano Statale, Milan, Italy

**Keywords:** neurofeedback, dyslexia, reading, training, learning disabilities (LD)

## Abstract

Neurofunctional models of developmental dyslexia (DD) point out disruption of the left-lateralized reading network. In individuals with DD, the left temporo-parietal (TP) regions are underactivated during reading tasks and a dysfunctional activation of the contralateral regions is reported. After a successful reading intervention, left TP lateralization was found to be increased in children with DD. Previous studies measured the effect of modulating the excitability of the left TP cortex using non-invasive brain stimulation (NIBS) in individuals with reading difficulties, showing significant reading improvements. NIBS exclusion criteria and safety guidelines may limit its application in settings without medical supervision and in younger populations. Neurofeedback (NF) training could be an alternative intervention method for modulating the inter-hemispheric balance of the temporal–parietal regions in DD. To date, the effect of NF on reading has been scarcely investigated. Few protocols increasing beta activity in underactivated areas showed improved reading outcomes. However, none of the previous studies designed the NF intervention based on a neurofunctional model of DD. We aim to propose a study protocol for testing the efficacy of a NF training specifically designed for inducing a functional hemispheric imbalance of the tempo-parietal regions in adults with DD. A randomized clinical trial aimed at comparing two experimental conditions is described: (a) Enhancing left beta/theta power ratio NF training in combination with reducing right beta/theta power ratio NF training and (b) sham NF training.

**Clinical Trial Registration:** www.ClinicalTrials.gov, identifier [NCT04989088].

## Introduction

Developmental dyslexia (DD) is a neurodevelopmental disorder associated with a persistent impairment in the acquisition and development of reading. Diagnostic criteria include a performance in reading speed and/or accuracy that is significantly below the norm, despite average intelligence, adequate educational opportunities, and secure socioeconomic conditions (World Health Organization, 1992; American Psychiatric Association, 2013). Due to their interferences with academic achievement, DD-related difficulties are often associated with low self-efficacy (Lackaye and Margalit, 2006; Magenes et al., 2021) and can ultimately lead to emotional and behavioral problems (Capozzi et al., 2008). At a neurocognitive level, the impaired grapheme–phoneme conversion mechanism is assumed to be based on a dysfunctional letter-speech sound integration (Richlan, 2019), associated with specifically altered brain activations.

### Neurofunctional Models of Dyslexia

Over the past two decades, cross-linguistic neuroscientific research converged on a functional neuroanatomical model of DD (Richlan, 2014). In particular, altered brain activation in readers with DD was consistently identified in the left-sided reading network (Pugh et al., 2000, 2010; Démonet et al., 2004; Shaywitz and Shaywitz, 2005). In adult typical readers, the reading system includes three circuits, namely, two posterior pathways (i.e., dorsal and ventral) processing visual and orthographic information and one anterior component connected to them. The dorsal pathway, including mainly the angular and supramarginalis gyri (Price, 1998), is involved in phonology-based decoding. The ventral pathway is centered in the posterior fusiform gyrus and represents automatic access to the visual word form area (VWFA) (Cohen et al., 2002). The anterior component is located in the left inferior-frontal gyrus (Price et al., 2001) and is implicated in the output of phonological and articulatory aspects.

The standard neurofunctional model of DD posits three activation abnormalities in the reading network, and more precisely: (1) the reduced activity of a left dorsal temporo-parietal (TP) region, including the posterior aspect of the superior temporal gyrus and adjacent parietal regions, underpinning the phonological decoding difficulty; (2) the reduced activity of a left ventral occipito-temporal (OT) region, including lateral extrastriate, fusiform, and inferior temporal regions, associated with the difficulty in fast orthographic word recognition; and (3) the overactivation of the left inferior frontal gyrus (IFG), presumably reflecting a compensatory reliance on effortful articulatory processes.

A meta-analysis of neuroimaging studies conducted about DD (Richlan et al., 2009), aimed at evaluating the aforementioned standard model, confirmed the underactivation in left TP regions, specifically in the left inferior parietal lobule (IPL), superior temporal, middle temporal, inferior temporal, and fusiform regions. In contrast to the standard model, the meta-analysis pointed out the underactivation in a left IFG region associated with lexical and sub-lexical phonological representations access, together with the overactivation in the primary motor cortex and the anterior insula, serving as a compensatory reliance on articulatory-based phonological processes. The IPL abnormality was interpreted by the authors of the meta-analysis as part of a fronto-parietal attention network, interacting with reading processes *via* top-down connections.

To better explore the developmental trajectory of these abnormalities, a second meta-analysis by the same authors (Richlan et al., 2011) considered separately neuroimaging studies carried out on children and adults. Interestingly, the left TP underactivation was found only in adults but not in children, whereas the OT dysfunction was found in both populations, and more extensively in adults. Therefore, the authors suggested that an early small left OT dysfunction becomes increasingly extended during development and is later accompanied by a left TP dysfunction. These findings led the authors to posit a new neurofunctional model of DD (Richlan, 2012), including abnormalities in OT, IFG, and IPL regions. According to this model, the left IFG underactivation reflects the deficit in accessing lexical and sublexical phonological representations, whereas the left OT underactivation reflects a deficit in both words and pseudo-word processing, which becomes even more evident with increasing demands on phonological decoding.

An fMRI meta-analysis by Paulesu et al. (2014) (1) confirmed the underactivation of a left-lateralized network including the inferior frontal, premotor, supramarginal cortices, and the infero-temporal and fusiform regions and (2) excluded a specific activation of the left OT cortex in DD during reading and reading-like tasks.

Besides the left hemisphere underactivation, a dysfunctional increased right activation during reading tasks has been reported in individuals with DD (Shaywitz et al., 1998; Sarkari et al., 2002; Eden et al., 2004; Simos et al., 2007; Rimrodt et al., 2009). Although less consistent than the reduced left-sided activation, the right hemisphere overactivation was identified starting from the early phases of reading development, as in second-grade students (Bach et al., 2010), and has been interpreted as the effect of a compensatory activity during reading. Coherently, neuroimaging studies concerning intervention addressed to DD showed a reduction of right temporal activation after a successful reading intervention in children with DD (Shaywitz et al., 2004) and greater activation of the same area in children with DD who did not show reading improvements after a cognitive intervention (Odegard et al., 2008).

### Neuromodulatory Intervention for Dyslexia

The neuro-functional reorganization following an intervention addressing DD in those who exhibited a reading improvement (Eden et al., 2004; Shaywitz et al., 2004; Odegard et al., 2008; Barquero et al., 2014) inspired the possibility to use non-invasive brain stimulation (NIBS) to modulate reading performance in individuals with DD (Krause and Cohen Kadosh, 2013; Vicario and Nitsche, 2013). A systematic review by Cancer and Antonietti (2018b) discussed the findings of NIBS studies on the effect of excitability alterations in the underactivated brain regions in DD. Increased excitability of the left TP cortex using transcranial direct current stimulation (tDCS) was the most frequently investigated intervention, showing consistent positive results on reading performance in adults (Turkeltaub et al., 2012; Younger et al., 2016) and children (Costanzo et al., 2016a,b, 2019) populations. Furthermore, the neuromodulation protocols investigated by Turkeltaub et al. (2012) and Costanzo et al. (2016a,b, 2019) included the conjunction of left TP excitability and the inhibition of the right contralateral cortex. Thomson et al. (2015) attempted to measure the specific contribution of monolateral vs. bilateral stimulation in typical readers. However, findings on healthy participants showed an opposite trend as compared to the studies on individuals with DD. The authors speculated that the results of neuromodulatory intervention designed to tap into the specific brain anomalies of the brain in individuals with DD could not be replicated in healthy individuals. Further empirical evidence is needed to confirm the specific effect of the bilateral NIBS intervention.

Although its promising effect in DD, NIBS exclusion criteria and safety guidelines may limit its application in settings without medical supervision and in younger populations. The effect and safety of NIBS in children were recently reviewed by Finisguerra et al. (2019). Findings support the safety of the practice; However, long-term responses of the immature brain to stimulation should be further investigated (Cohen Kadosh, 2013; Costanzo et al., 2016a). To date, no agreement has been reached in the clinical community about the ethical aspects of using NIBS in stimulating pediatric populations (Cancer et al., 2021).

### Dyslexia and Neurofeedback

One of the most promising non-invasive methods to support individuals with DD is EEG-based neurofeedback (NF), which can encourage desirable brain activity and reduce dysfunctional brain activity through implicit mechanisms related to operant conditioning. First, EEG electrodes are placed on the scalp. The brain activity is recorded by dedicated software that analyzes it and, according to specific parameters of interest, returns feedback that can be either visual, auditory, or better, both (as a videogame). The users are familiarized with the interface and are accompanied by a professional who can provide some hints to facilitate the procedure. When the brainwaves get close to the set parameters, the users receive positive reinforcement (Au et al., 2014). This way, it is possible to create proficient and functional associations with different psychophysiological states. Such associations can more easily arise if the feedback is provided very quickly. That is why EEG has been the election choice so far. In the near majority of cases of DD-related applications, the users are children. Thus, the simpler and more fun the interface, the better. Finally, NF can also be proposed offline, for example before beginning the session to induce the ideal psychophysiological state, as well as while performing some specific exercises.

In the following paragraphs, we will briefly describe the available literature on the topic encouraging a discussion on the choices that have been made about NF training, such as the brainwaves parameters, the brain region of interest, and the type of feedback.

One of the most famous and applied NF protocols is Lubar’s one, which was tailor-made for ADHD patients. Starting from the idea that ADHD and learning disabilities present a high level of comorbidity and share some neurofunctional mechanisms, the protocol has been extended also to users with DD. The principle is to encourage β frequencies and to decrease θ ones (the so-called β/θ ratio). These kinds of training are mainly targeted at reinforcing attentional skills and visuo-motor integration (Au et al., 2014). However, with specific regard to reading abilities, it has been shown that this protocol applied with a montage over either C3 or C4 can influence visual word recognition (Barnea et al., 2005). One previous study applied this technique to explore its feasibility with the Chinese language in a pilot sample of 4 children (Au et al., 2014). The training included both unipolar protocols, intending to increase β power over C3 site, and bipolar montage involving C3 and C4, to optimize interhemispheric functional connectivity and sensorimotor integration between C3 and C4. Along with the neurophysiological recording of the different brainwave frequencies, a neuropsychological assessment was conducted before and after the NF training. The pre–post within-subject comparison revealed that the NF training increased the β/θ ratios in all participants and improved the performance in the tasks for auditory vigilance and phonological awareness.

The same protocol with β/θ ratios was adopted by Sadeghi and Nazari (2015) in a combined ERP-NF study also focused on visuo-spatial attention. The authors in this case placed the electrodes over O1 and O2 to target a most posterior network and were able to observe positive effects of the training at a behavioral level: the participants showed a reduction in the reaction times and an increase in the correct responses. Such improvement was accompanied by physiological changes, such as P3a and P3b amplitude reduction, which can be interpreted by postulating a decreased attentional demand at the neural level.

Interestingly, Nazari et al. (2012) used a similar protocol with a training set to decrease δ and θ and to increase β at T3 and F7 taking into account the double-deficit theory of DD (Arns et al., 2007). However, in this case, the authors directly explored the effects of the training over more specific linguistic skills, such as phonological awareness and reading abilities. The results of this study highlighted a significant improvement in these outcomes along with a normalization of coherence of the band power in different couples of electrodes. According to the authors, these kinds of changes could indicate integration of sensory and motor areas that could explain the behavioral advantage. This approach was also applied by Raesi et al. (2017), who found significant improvements in accuracy, comprehension, and spelling after the training.

More recently Eroğlu et al. (2020) published a research paper with two main important features. First, they used multiscale entropy analysis (MSE) for measuring dynamical complexity in the temporal features of the brainwaves. Second, they applied a protocol based on a more individualized approach. Indeed, the NF training was adjusted over the EEG activity of the participants. From here on, the NF setup was aimed at: (1) reducing theta waves at Broca area if above the threshold; (2) reducing theta waves at Wernicke area if above the threshold; (3) finding the channels with the maximum absolute power of theta waves at the left hemisphere and reduce it; and (4) finding the channels with the maximum absolute power of theta waves at the right hemisphere and reduce it. Although they did not consider behavioral outcomes, the researchers were able to observe that, after the training, the lower complexity of participants with DD increased to the typically developing group’s levels.

Finally, Breteler et al. (2012) designed a treatment that was still personalized to each individual but considering the presence of hypocoherence between couples of channels. Data analysis showed that the most common connections found to be too low were between occipital–parietal and frontal–temporal regions and parietal to medial temporal connections. In this case, the authors also considered behavioral outcomes for reading abilities. Results underlined that, after the treatment, the experimental group improved its reading scores.

To the best of our knowledge, despite the significant results obtained in previous NF research, no study has investigated a bilateral NF intervention targeting TP activation. The conjunction effect of the left increased and right decreased TP activation was found to be effective in NIBS interventions for Turkeltaub et al. (2012); Costanzo et al. (2016a, b); Younger et al. (2016).

Our hypothesis is to induce a reading performance improvement using bilateral NF to restore the typical TP hemispheric imbalance in the brain (Shaywitz et al., 1998; Sarkari et al., 2002; Eden et al., 2004; Simos et al., 2007; Rimrodt et al., 2009). As compared to NIBS, NF has fewer exclusion criteria and limitations in its application and could be safely applied in pediatric populations.

The present study protocol was designed to test the effect of a single-session bilateral NF protocol aimed at increasing the β/θ ratio in left TP areas and decreasing the β/θ ratio in the contralateral area on bilateral TP hemispheric imbalance during linguistic tasks in adults with DD. The second purpose of the study is to explore whether and to what extent the degree of asymmetry in the β/θ ratio can influence the outcomes of the training. Finally, the study aims at exploring the effect of the novel NF protocol on the reading and phonological skills of participants.

## Methods and Analyses

### Design

To test the effectiveness of a bilateral NF TP protocol for improving reading in adults with DD, a randomized controlled trial was designed. More precisely, participants will be randomly allocated to one of two parallel conditions, namely (a) the experimental condition (i.e., enhancing left β/θ and reducing right β/θ NF training); or (b) the placebo comparator (i.e., sham NF training). The trial protocol was registered on Clinicaltrials.gov (Clinical Trial ID: NCT04989088).

### Participants

Forty participants will be enrolled in the study. Eligibility of participants will be checked based on the following inclusion criteria: (a) adults aged 18–35 years; (b) to have a diagnosis of DD (ICD-10 code: F81.0) (World Health Organization, 1992) or a reading performance of at least 1.5 SD below the norm. Exclusion criteria include intellectual disability, psychiatric conditions, comorbidity with neurodevelopmental disorders (e.g., ADHD), neurological disorders, epilepsy, and enrollment in linguistic or literature university courses. Participants will be recruited by advertising the study through the Learning Disability services of two university campuses. Potential participants will have to provide demographic, clinical (i.e., psychiatric and neurological conditions), and educational information (i.e., years of education, field of study, average grades) and to complete a few self-report standardized questionnaires about their reading abilities. Potential participants who received a diagnosis of DD made by an independent clinician prior the enrollment in the investigation will be asked to share their clinical documentation with the researchers.

### Procedure

Participants who will respond to the recruitment advertisement will receive a detailed description of the experimental procedure and written informed consent to participate in the study *via* email. If they will provide their consent, participants will be asked to complete an online screening phase to check their eligibility. Eligible participants will be selected based on the inclusion and exclusion criteria of the study and will be randomly assigned to one of two experimental conditions (i.e., active NF training; sham NF training; and 1:1 ratio).

The study will include two experimental sessions: A preliminary screening session will be conducted to record EEG data of participants during resting state (i.e., eyes open 3-min) and two linguistic tasks (see section “Neurophysiological Measures”). This phase will provide information about EEG band variability. Data of participants who will have shown an abnormally low α frequency (i.e., α peak frequency below about 8.5 Hz at TP during resting-state), which could potentially make calculations on the β/θ ratio misleading, will be excluded from the analyses. EEG data collected during the screening phase will be elaborated; A measure of hemispheric asymmetry will be calculated (i.e., TP7–TP8) to (1) monitor whether and to what extent the degree of asymmetry in the ratio can influence the outcomes of the following training, and (2) to allocate participants equally to the experimental and the placebo conditions. Indeed, participants will be assigned to one of two experimental conditions (i.e., active NF training; sham NF training; and 1:1 ratio) using stratified randomization based on the β/θ asymmetry scores.

Thereafter, during the second experimental session (NF training), each participant will be asked to participate in one lab session of approximately 90 min, which will include seven phases: (1) pre-training behavioral assessment using standardized tests to measure reading, rapid automatized naming (RAN), and verbal working memory; (2) pre-training EEG recording as proposed in the screening session (resting state and the two linguistic tasks randomized to avoid familiarity effects; (3) NF warm-up (NF1; 10 min); (4) NF training (NF2; 20 min); (5) post-training behavioral assessment using the same tests of the pre-training phase; (6) NF reinforcement training (NF3; 10 min); and (7) post-training EEG recording. Time breaks will be provided to exclude excessive cognitive workload and fatigue during the procedure. Finally, to test the appropriateness of the blinding procedure, participants will be asked to judge their NF condition allocation, by reporting if they think they received either the sham or the active NF training.

### Behavioral Measures

Behavioral assessment includes three self-report questionnaires for reading problem screening, namely the Revised Adult Dyslexia Checklist (Vinegard, 1994), the Adult Reading Questionnaire (ARQ) (Snowling et al., 2012), and the Adult Reading History Questionnaire (ARHQ) (Lefly and Pennington, 2000). Such questionnaires measure the respondent’s academic achievements, attitude toward school, perceived reading and writing difficulties, reading and writing habits and preferences, time management, and organization problems.

To measure reading, standardized norm-referenced text, word, and pseudo-word reading tasks from the LSC-SUA Battery (Montesano et al., 2020) are used. Participants will be asked to read aloud a text passage and lists of words and pseudo-words. For each reading task, reading speed (i.e., syllable/second) and reading accuracy (i.e., number of errors) will be recorded.

Besides reading, RAN is assessed using the RAN test (De Luca et al., 2005), which was found to be a strong predictor of reading difficulties in older individuals (Cancer and Antonietti, 2018a). In this test, participants are required to sequentially name various visual stimuli (i.e., colored squares, black and white icons, and numbers) presented in 10 × 5 matrices. Naming speed (expressed in seconds) and naming accuracy (expressed in the number of naming errors) are recorded.

Finally, verbal working memory, an ability that is typically impaired in individuals with DD (Toffalini et al., 2017), is assessed using the forward and backward digit span task (Wechsler, 2008).

### Neurophysiological Measures

EEG cortical activity will be recorded using the ProComp5 Infiniti System paired with the BioGraph Infiniti Software (Thought Technology Ltd.) from two active sites placed in TP7 and TP8, according to the International 10–10 system (Nuwer et al., 1998). Two reference electrodes will be placed on the earlobes and two ground electrodes will be placed on the mastoids. The sampling rate will be set at 256 Hz and impedance will be kept below 5 kΩ. Artifact rejection will be performed using both an amplitude threshold (20 μV) and visual inspection.

During the screening session, a measure of hemispheric asymmetry will be calculated by subtracting the right β/θ power ratio from the left β/θ power ratio at TP (i.e., TP7–TP8).

For the NF session, the separate power of the α frequency (8–12 Hz), the δ frequency (1–3 Hz), along with that of the target frequencies (β: 13–21 Hz; θ: 4–8 Hz) and their ratio (β/θ) will be extracted by the BioGraph Infiniti Software and will be considered for the analyses. Pre and post-training EEG recordings will include three phases: (1) resting-state with eyes open for 3 min; (2) a computerized phonological task, in which participants are required to decide whether a list of bi-syllabic word pairs rhymed or not (Spironelli et al., 2006, 2008); and (3) a computerized semantic task, in which participants are required to decide whether bi-syllabic word pairs from a list were semantically related or not. For a detailed description of the linguistic tasks, see Spironelli et al. (2006, 2008). For phases (2) and (3), both response times and accuracy rates will be recorded.

### Neurofeedback Protocol

The NF intervention will consist of three NF sessions using the ProComp5 Infiniti paired with the BioGraph Infiniti Software (Thought Technology Ltd.), specifically: (1) a NF warm-up session of 10 min (NF1); (2) a NF training session of 20 min (NF2); and (3) a NF reinforcement session of 10 min (NF3). The NF training will target cortical β/θ activity in the left TP area (TP7) and β/θ activity in the right TP area (TP8), so to suppress the θ activity and enhance the β activity in the left hemisphere, while suppressing β activity and enhancing the θ activity in the right hemisphere. Rewards thresholds adjust themselves automatically and are maintained in a position where the feedback is given 80% of the time. During NF, participants will be sitting comfortably in a chair to reduce muscle tension, in front of a 27″ monitor. A mini-game (“Bust Big Balloons”) from Zukor’s Carnival feedback videogame (Zukor Interactive) will be used. The reward condition will inform participants of increased left β/θ and decreased right β/θ, through animations and sounds representing successful balloon busting (the balloons will burst sequentially from left to right). The inhibit condition will inform participants of decreased left β/θ and increased right β/θ and will cause a visual distractor to appear on the screen (i.e., flickering lights covering the screen) and the balloon busting will stop. Whenever participants can maintain the amplitude of left EEG β/θ ratio above the threshold while maintaining the amplitude of the right θ/β ratio above the threshold for at least 2 s, feedbacks will be given. During training, separate metrics (i.e., β, θ, and β/θ) for each hemisphere will be recorded; Both left and right β/θ will need to meet the threshold to receive the feedback.

Before the main NF training session, a short warm-up session will be delivered to introduce the task using a simplified feedback mechanism. More precisely, the flickering lights animation will be employed for both reward and inhibit feedbacks. Following the main NF training session, a reinforcing session will be delivered to induce cognitive and neurophysiological changes that will last for the extensive length of both post-training behavioral assessment and EEG recording phases – see for example the protocol by Agnoli et al. (2018) for creative enhancement.

As for the placebo condition, a sham NF training will be delivered by showing to control participants pre-recorded video clips of the game animations for the duration of the NF sessions, following the same procedure of the experimental condition (Egner et al., 2002; Ros et al., 2013; Hosseini et al., 2016; Agnoli et al., 2018). Reward and inhibit feedbacks will correspond to a pre-recorded NF demonstrative session, and therefore will not be related to the actual amplitude variation of the bilateral β/θ ratios.

### Data Analyses

First, demographics and behavioral baseline assessment of experimental and control groups will be compared, to test any potential difference between groups for variables relevant to the aim of the study, together with the variables related to the asymmetry scores as described in section “Procedure”. Thereafter, a series of GLMs is intended to be carried out to compare the effect of the active NF training with those of the sham NF training. For doing that, a mixed factorial ANOVA 2 × 2, considering Phase (pre vs. post) and Condition (experimental vs. sham) as the independent variables will be carried out for each dependent measure (i.e., separate left and right β and θ frequencies power during the linguistic tasks; left β/θ and right β/θ EEG power in resting state and during the linguistic tasks; accuracy and reaction times during the linguistic tasks, reading task, and RAN; accuracy in the digit span task). A correction for multiple comparisons will be applied (i.e., the Bonferroni correction). A sample size of 40 was calculated to be enough to achieve a statistical power of 0.87, for medium effect size (η^2^ = 0.06), by setting alpha at 0.05, in the GLMs we planned to carry out to test the effectiveness of the NF intervention.

## Expected Results

According to our hypothesis, the experimental single-session bilateral TP NF protocol (enhancing left β/θ and reducing right β/θ) would restore the typical reading-related inter-hemispheric imbalance in adults with DD. Accordingly, we expect to find an enhanced left β/θ power ratio and a concurrent reduced right β/θ power ratio during the performance of linguistic tasks after the active NF training. Conversely, we expect the control group (i.e., placebo NF training) to maintain an abnormal activation of TP regions during the linguistic tasks (i.e., reduced left β/θ and enhanced right β/θ) and no modulation of the β/θ power ratio after training. In summary, we expect a significant Phase (pre vs. post) × Condition (active NF vs. placebo NF) interaction effect on the modulation of bilateral TP imbalance. Also, we expect these improvements to be greater for those participants with larger asymmetry scores as assessed in the screening phase.

As for the behavioral measures (i.e., reading, RAN, and verbal WM), despite previous evidence on cognitive enhancement after a single-session NF (e.g., Escolano et al., 2014), we did not formulate a specific hypothesis, but instead, we opted for an exploratory approach, due to the potential limited effect size of a novel single-session NF protocol in DD.

## Discussion

Despite the current limited number of NF protocols for DD, previous evidence showed promising effects of NF training in inducing beneficial changes in reading-related functions (Breteler et al., 2012; Au et al., 2014; Sadeghi and Nazari, 2015; Eroğlu et al., 2020). To date, no previous study investigated a bilateral NF intervention targeting TP activation. Such novel bilateral NF intervention was adapted from effective NIBS studies (Turkeltaub et al., 2012; Costanzo et al., 2016a,b; Younger et al., 2016).

As compared to NIBS, NF has fewer exclusion criteria (Marzbani et al., 2016) and it is commonly used on neurodevelopmental disorders across the lifespan in clinical settings (e.g., on ADHD patients; Holtmann et al., 2014), fewer concerns about safety and ethical matters. Therefore, bilateral NF could be a more easily applicable neuromodulatory option for both younger and older individuals with DD.

In addition to proposing a novel NF intervention protocol, the present study protocol introduces a methodological advantage, namely, a placebo NF condition. Differently than previous NF experiments which used no-training control conditions in which participants were just resting, with this protocol we will propose a more controlled placebo condition where participants from both groups will receive the same instructions and the same visual experience. Indeed, the only difference will rely on the fact that the videogame would not be linked to the brain activities of control participants, as already proposed by other authors (Egner et al., 2002; Ros et al., 2013; Hosseini et al., 2016; Agnoli et al., 2018). This procedure will prevent some confounding effects related to unbalanced conditions in terms of cognitive effort, visual-perceptual stimulation, and motivation. Also, in this protocol, we opted for a single fake video (placebo) for all control participants to limit further confounding effects related to inter-subjective variability. Furthermore, compared to an alternative NF protocol (e.g., targeting different power frequencies and/or different brain regions), the placebo NF could help isolate the specific effect of the experimental NF mechanisms, and thus draw clear conclusions about its functional effect.

Another methodological novelty of the present protocol is the use of the β/θ power ratio in NF intervention for DD to suppress the θ activity and enhancing the β activity in the left TP regions while enhancing the θ/β power ratio in the less involved right TP regions during reading. The θ/β power ratio (Lubar, 1991) was considered based on its functional relevance as a marker of central nervous system arousal (Mann et al., 1992) and its prognostic value for neurodevelopmental disorders (Arns et al., 2013), as demonstrated by numerous findings on effective θ/β NF training in ADHD (Holtmann et al., 2014). The use of power-ratio metrics could be potentially associated with confounds and difficulties in their interpretations, as changes in ratio could stem from changes in any of the frequency bands. Furthermore, β and θ changes following NF are not always consistent (Janssen et al., 2017). To avoid this potential issue, each frequency band will be separately evaluated during the training periods, to extract the key driver of the ratio modulation effect.

As for the NF game selection, we opted for gameplay consisting of a matrix of visual stimuli (i.e., balloons) exploding sequentially from left to right, thus mirroring the typical visual scanning required during reading tasks. We hypothesize that such eye movements during the NF training would produce a matching neuropsychological condition that would facilitate the modulation of the reading-related TP imbalance.

Another important issue to discuss refers to our choice to use a single session in the light of conventional NF training studies, which include many consecutive sessions. Indeed, although the presence of positive effects has been mainly reported for longer protocols, the present single-session protocol aimed to propose a theoretical and practical model for NF interventions in DD to test whether a one-shot training could be sufficient to obtain some observable improvement on the behavioral but, above all, on the neurophysiological level. If so, the results could provide further information to plan longer-term sessions. Thus, our protocol explores the feasibility to target a well-known neurofunctional model, which, however, is rather neglected by the NF literature. Also, the clinical implications of such intervention will be taken into account for further developments.

A limitation of the present study protocol is the use of a low-resolution two-channel EEG. This EEG setup was selected, instead of a higher-resolution setup, for both practical reasons (it is the equipment which is most widely used to deliver treatments), and for studying the specific locations which are the object of the present protocol, namely bilateral TP, which are particularly relevant for DD neurofunctional basis.

Despite the discussed limitations, the present study protocol would be the first attempt to adapt the neuromodulation hypothesis of DD for NF training by testing both the feasibility and efficacy of a novel rehabilitation procedure for DD, and would finally lay the groundwork for future NF applications for DD.

## Ethics Statement

Ethical approval was obtained by the Psychology Research Ethics Committee of the Università Cattolica del Sacro Cuore, Milan, Italy on the 22nd January 2021 (Approval Numbers: 11-21). Participants will provide their written informed consent to participate in the study.

## Author Contributions

AC and AA conceived the theoretical framework. AC, MV, and CL conceived and designed the neurofeedback training. MV and CL supervised the EEG methods. AC and MV performed the pilot experiments to test the procedures and wrote the first draft of the manuscript. AA and CL supervised the project. AC, MV, CL, and AA wrote the final version of the manuscript. All authors contributed to the article and approved the submitted version.

## Conflict of Interest

The authors declare that the research was conducted in the absence of any commercial or financial relationships that could be construed as a potential conflict of interest.

## Publisher’s Note

All claims expressed in this article are solely those of the authors and do not necessarily represent those of their affiliated organizations, or those of the publisher, the editors and the reviewers. Any product that may be evaluated in this article, or claim that may be made by its manufacturer, is not guaranteed or endorsed by the publisher.
